# Leptospirosis in Southeast Asia: Investigating Seroprevalence, Transmission Patterns, and Diagnostic Challenges

**DOI:** 10.3390/tropicalmed11010018

**Published:** 2026-01-07

**Authors:** Chembie A. Almazar, Yvette B. Montala, Windell L. Rivera

**Affiliations:** Pathogen-Host-Environment Interactions Research Laboratory, Institute of Biology, College of Science, University of the Philippines Diliman, Quezon City 1101, Philippines; caalmazar@up.edu.ph (C.A.A.); ybmontala@up.edu.ph (Y.B.M.)

**Keywords:** Southeast Asia, leptospirosis, seroprevalence, disease transmission, environmental factors, spatiotemporal dynamics, disease surveillance, diagnostics

## Abstract

Leptospirosis remains a significant public health and economic burden in Southeast Asia, particularly in low- and middle-income countries where environmental, occupational, and socioeconomic factors contribute to its endemicity. Transmission is driven by close interactions between humans and infected animal reservoirs, alongside climatic conditions such as heavy rainfall and flooding. The region’s high but variable seroprevalence reflects inconsistencies in diagnostic methodologies and surveillance systems, complicating disease burden estimation. Major gaps persist in diagnostic capabilities, with current tools often unsuitable for resource-limited settings, leading to underdiagnosis and delayed treatment. Environmental modeling and spatial epidemiology are underutilized due to limited interdisciplinary data integration and predictive capacity. Addressing these challenges requires a One Health approach that integrates human, animal, and environmental health sectors. Key policy recommendations include harmonized surveillance, standardized and validated diagnostics, expanded vaccination programs, improved animal husbandry, and targeted public education. Urban infrastructure improvements and early warning systems are also critical, particularly in disaster-prone areas. Strengthened governance, cross-sectoral collaboration, and investment in research and innovation are essential for sustainable leptospirosis control. Implementing these measures will enhance preparedness, reduce disease transmission, and contribute to improved public health outcomes in all sectors across the region.

## 1. Introduction

Leptospirosis is one of the most common and the most widespread of zoonotic diseases. The economic burden of leptospirosis remains underestimated due to underreporting, diagnostic limitations, and data inconsistencies. The initiative to include leptospirosis on the index of Global Burden of Disease, which was not formalized until 2010, allowed the estimation of its burden through disability-adjusted life years (DALYs) [1]. The WHO Leptospirosis Burden Epidemiology Reference Group (LERG) standardized global metrics using DALYs to quantify mortality and morbidity impacts [2]. This metric measures the burden of disease that captures both the premature mortality as well as the prevalence and severity of ill-health. In 2015, it was estimated that approximately 2.90 million DALYs were lost per annum from 1.03 million cases reported previously, which puts the disease at about 73% burden that of cholera [3].

The latest data on the global productivity cost of leptospirosis in DALYs, factoring in the Gross Domestic Product (GDP), were measured in 2019, indicating it to be at USD 29.3 billion with low and high estimates at USD 11.6 billion to USD 52.3 billion [4]. While China, India, Indonesia, Sri Lanka, and the United States have the highest productivity losses due to leptospirosis, eight out of ten countries with the highest burden were in the Asia-Pacific region. The Southeast Asia region shares 38.9% of the total global burden of the disease, while 47.1% accounts for 53 of the lower middle-income countries and territories, indicating that the disease is much of an economic problem as it is a public health problem. High-burden countries, including Thailand, Vietnam, Indonesia, and Malaysia, each have a productivity loss estimate of more than USD 500 billion due to leptospirosis [4]. In the Philippines alone, the burden of the disease was estimated to be at USD 124.97 million per year, which accounts for 1.13% of Metro Manila’s GDP [5].

Furthermore, the direct economic impact of leptospirosis was estimated to be USD 473 per patient, which includes the sum of the costs of hospital stay, diagnostic procedures, therapeutic management, and median income losses due to absence from work of both the patient and the caregiver [6]. Loss of productivity was also reported in Banyumas District, Indonesia at USD $289.64 per patient, accumulating a yearly productivity loss value estimate at USD 388,499, proving its direct and indirect economic toll on populations where the disease is endemic [7]. On the other hand, a cross-sectional willingness-to-contribute (WTC) model was performed for Kelantan, Malaysia, to quantify the cost of preventing leptospirosis which revealed that between USD 106.7 million to USD 315 million per annum can be saved through the comprehensive prevention of the disease [8]. The significant overall economic burden of leptospirosis, therefore, demands its inclusion in the global health agenda for comprehensive disease control and prevention efforts.

Leptospirosis in Southeast Asian countries is endemic [9,10]. The endemicity of this disease in this region is brought about by several factors that encourage its growth, proliferation, and, hence, transmission from host reservoirs to human populations. Leptospirosis remains to be one of the most widespread zoonotic diseases. The disease caused by the pathogenic *Leptospira* species is prevalent in tropical and subtropical regions with a high risk of infection to those vulnerable individuals exposed to contaminated environments and animal host reservoirs [11]. The disease primarily affects vulnerable populations in the urban areas in developing countries most affected by heavy rainfall and flooding as well as rural farmers and those involved in water recreational activities. Since animals are the main reservoir of *Leptospira*, humans can acquire the infection through percutaneous contact of contaminated water or soil with the urine of infected animals. The complex transmission of the pathogen involves more than 60 species of *Leptospira*, a wide range of reservoir animals, and ecological conditions in tropical countries with abundant exposure to soil and water make it extremely difficult to control leptospirosis [10].

The objective of this review is to summarize current efforts in pathogen surveillance in host reservoirs and the role of the environment that highlights the importance of understanding disease cycle and its transmission in the Southeast Asian region where the disease is endemic. It also focuses on studies on the incidence of infection among human and host populations across the region as well as recent studies on spatiotemporal dynamics of the disease that are vital in determining disease incidence and transmission. Finally, the review addresses current efforts in leptospirosis diagnostics for effective disease surveillance and mitigation, especially in high-risk and vulnerable populations. This review highlights several research gaps that could be potentially explored by researchers to achieve sustainable solutions for leptospirosis prevention and control.

## 2. Seroprevalence of Leptospirosis in Southeast Asia and Factors Affecting Disease Transmission

### 2.1. Leptospira Seroprevalence in Human Populations

Southeast Asia is a leptospirosis global hotspot, with high modelled incidence and frequent monsoon and flood-associated outbreaks. Thailand experienced a marked incidence surge in 2000 linked to flooding [12], while Indonesia reports repeated flood-related outbreaks and notable case fatality in some events [13]. Meanwhile, Sri Lanka exhibits high hospitalization incidence with clear rainy season peaks [14], while the Philippines reports large flood-associated outbreaks in urban areas with poor sanitation [15]. Compared to other regions, Southeast Asia may be comparable to Oceania in terms of incidence in some island settings, while both the Latin America and the Caribbean frequently report large urban post flood outbreaks. Sub-Saharan Africa likely has high burden but sparse incidence data while Europe and North America have low endemic incidence with spikes after floods or via recreational water exposures [16,17]. In the global setting, South and Southeast Asia had among the highest morbidity and mortality, alongside Oceania, Latin American subregions, and East Sub-Saharan Africa. Some incidence figures include Indonesia (~39 per 100,000) and India (~20 per 100,000), while Oceania (regional aggregate) showed very high incidence and mortality [18]. Local African estimates can also be high (e.g., northern Tanzania ~75–102 per 100,000), but data are sparse compared with Asia and Latin America. The region also bears a disproportionately high burden compared to Europe and North America as the disease is mostly associated with travel and the occasional post-flood spikes [18].

Available serological data indicate widespread exposure to *Leptospira* across Southeast Asia, with marked variation by country and study population (Table 1). Included in the above table are selected studies which measured human seroprevalence (IgG/IgM antibodies against *Leptospira* sp.) using MAT, ELISA, and rapid tests. The general population and risk groups in Southeast Asia (Brunei, Cambodia, Indonesia, Laos, Malaysia, Myanmar, Philippines, Singapore, Thailand, Timor-Leste, Vietnam) including community and blood donor serosurveys, household surveys, and occupational cohorts are also included. Extract period (year), assay type and cut-off (e.g., MAT ≥ 1:100), population, and point/pooled estimates were also identified. Exclusion criteria are applied to incidence-only studies, animal studies, and non-SE Asia regions.

Community and cohort data from Cambodia, Vietnam, and Lao PDR suggest non-trivial background exposure in rural/agricultural settings. Cambodian children in community fever surveillance conducted from 2007 to 2009 exhibited substantial IgM seroconversion on paired sera. However, only a minority were MAT confirmed at ≥1:100, and the reported risk suggested that ~1% of fevers per semester were due to leptospirosis [19]. Vietnamese community studies report child IgG seroprevalence around 13% and adult MAT seroprevalence in the Mekong Delta ranging roughly 11–21%, whereas rural Lao adults demonstrated ~24% MAT seropositivity in cited studies [20,21,22,23]. In contrast, the low seropositivity among Thai blood donors (1.7% by IgG ELISA; no recent infection by MAT) likely reflects a healthier, lower-exposure sampling frame [24]. Meanwhile, high-risk occupational cohorts demonstrate elevated exposure in specific settings, exemplified by 33.6% MAT seroprevalence among Malaysian wet market workers, while Metro Manila sewer workers had a more modest 4.8% (MAT ≥ 1:100) [27,28]. Indonesian reports collated in regional reviews show double-digit seropositivity in select surveys and flood-associated sampling, underscoring ecological and event-driven variability [16].

This observed heterogeneity in seroprevalence estimates is driven by assay choice, test cut-offs, timing of sampling, and laboratory panel composition. ELISA-IgM on convalescent or paired sera identifies recent exposure/seroconversion and tends to yield higher proportions than single-sample MAT; however, only a subset of ELISA seroconversions is MAT-confirmed at conservative cut-offs (≥1:100), as seen in Cambodia. Conversely, genus-level IgG ELISAs (e.g., in Thai donors) can detect low-level past exposure missed by MAT panels, especially if the live-serovar panel does not match local antigenic circulation. There is also a difference in thresholds on reported MAT cut-offs and serovar panels across various studies (e.g., single high titer vs. seroconversion; ≥1:50 vs. ≥1:100) that can meaningfully shift positivity. Timing relative to illness is also critical such that MAT seroconversion may lag, and acute samples alone cannot determine if an infection is present. Together, these factors constrain cross-study comparability and the feasibility of formal pooling without harmonization.

Sampling frames across the studies included in this review differ substantially. Healthy donors (Thailand) likely under-represent high-exposure agricultural or flood-prone populations, while occupational cohorts (Malaysia wet market workers; Philippines sewer workers) capture targeted risks but are not generalizable to the population at large. Community febrile cohorts (Cambodia) reflect incidence among children and identify recent infections but do not directly estimate cumulative exposure in general populations. Community adult MAT surveys (Vietnam, Lao PDR) better approximate background exposure but may still be biased by site selection and agricultural seasonality. Event-driven sampling, on the other hand (e.g., Indonesian flood-associated sampling), emphasizes outbreaks and ecological triggers, inflating estimates relative to inter-epidemic conditions.

Uneven geographic coverage can also be observed across the region. Robust, contemporary community serosurveys with harmonized assays were not identified for Singapore, Brunei, Myanmar, and Timor-Leste. This lack of regional syntheses acknowledges surveillance and publication gaps for several countries. Even in data-rich countries, temporal breadth is limited, with many estimates reflecting single time points or older studies, impeding trend analyses and the assessment of climate or land-use impacts over time. Within-country heterogeneity (urban vs. rural; flood-prone vs. highlands) is also infrequently mapped with sufficient resolution to inform subnational risk.

Given the assay and methodological design heterogeneity, cross-country comparisons can only be performed qualitatively. ELISA-based seroconversion data (Cambodia) and child/adult community MAT data (Vietnam, Lao PDR) collectively indicate endemic exposure in multiple settings, but numeric differences may stem as much from methods as from true epidemiology. Low seropositivity among healthy Thai donors does not contradict higher community or occupational estimates elsewhere but rather likely reflects selection and assay effects. Occupational data show that specific work and environmental contacts (wet markets, sewage) confer risk, but effect sizes vary across contexts. Older Indonesian surveys, on the other hand, illustrate that extreme weather and flooding amplify exposure, consistent with known drivers of leptospirosis infection.

Priorities for surveillance and research include standardizing serosurvey methodologies, including adopting agreed MAT panels reflecting local serovar circulation, harmonizing cut-offs (e.g., paired sera with rise to ≥1:100), reporting both single-sample and paired metrics, and pairing ELISA (IgM, IgG) with MAT to capture recent and past exposure. Researchers should also consider expanding representative sampling such as implementing nationally or provincially stratified community serosurveys covering rural/agricultural, peri-urban, and urban settings, with seasonal replication to capture hydrometeorological influences. There is also a need to fill geographic gaps that prioritize Singapore, Brunei, Myanmar, and Timor-Leste for baseline serosurveys and integrate with routine febrile illness diagnostics. It is also important to link serological evidence to leptospirosis incidence such that the combination of seroconversion cohorts (e.g., school-age children) with clinical and meteorological data that can calibrate incidence models and early warning systems for flood-associated risk. The implications for public health are considerable, as high seroprevalence in certain groups underscores the necessity for targeted interventions—ranging from the deployment of personal protective equipment among at-risk workers to the integration of leptospirosis surveillance into national health reporting systems. Ultimately, there is a pressing need for integrated One Health approaches that encompass human, animal, and environmental surveillance to align MAT panels with circulating serovars and elucidate transmission pathways, as well as for longitudinal studies that capture seasonal and geographic trends in disease transmission.

### 2.2. Animal Seroprevalence and the Maintenance of the Leptospira Transmission Cycle

Southeast Asia exhibits substantial animal exposure to *Leptospira* across livestock, companion animals, and rodents, but the magnitude and distribution of seroprevalence are markedly heterogeneous and highly assay dependent. In Thailand, a national cross sectional livestock survey 2001 found moderate seroprevalence in cattle (9.9%) and pigs (10.8%) but high levels in buffaloes (30.5%) using MAT at a ≥1:50 cut-off and a 24 serovar panel with predominant serovars being host associated (e.g., Ranarum/Sejroe/Mini in cattle; Mini/Sejroe/Bratislava in buffalo; Ranarum/Pomona/Bratislava in pigs) [32]. A later Thai passive surveillance dataset (2010–2015) using MAT at ≥1:100 reported higher cattle and buffalo seroprevalence (28.1% and 24.8%, respectively) and similar pig prevalence (11.3%), with Bratislava, Ranarum, Sejroe, Shermani, and Tarassovi frequently detected and multiple serovar reactions common, underscoring differences introduced by surveillance design and cut-off selection [25].

Vietnam’s recent multispecies survey in 2021 likewise illustrates broad exposure and serovar diversity. Specifically, 44.2% buffaloes, 32.9% dogs, 24.9% cattle, 16.0% rats, 12.2% cats, and 10.2% swine were MAT seropositive at ≥1:100 across a 25 serovar panel, with Hebdomadis, Patoc, Castellonis, and Javanica commonly detected [33]. A more stringent cut-off to ≥1:200 reduced positives and left Javanica as the predominant strain. Provincial variation (e.g., higher in Thai Binh) highlights micro ecological influences on exposure. In Malaysia, compiled evidence also reveals extreme subnational heterogeneity. Cattle seroprevalence ranging from very high in some provinces (e.g., Kelantan ~81.7% by MAT) to much lower at ~14% elsewhere. Porcine PCR positivity was ~6% in Selangor with Pomona. Dogs show low seroprevalence in working cohorts (≈3–6%) but high seroprevalence in shelter/clinical populations (up to ≈43% by MAT and PCR). Cats show values of ~15–26% by MAT. Rodents frequently demonstrate high renal PCR/culture positivity, consistent with a significant reservoir role [29]. Meta-analysis studies further confirm that pigs can range from single digits to >70% seropositive depending on country and method, cattle/buffalo are intermediate, dogs are typically moderate, and rodents are highly variable. Crucially, assay choice (MAT panel and cut-off) exerts a strong influence on apparent prevalence [34].

The scope of the studies in Table 2 includes research conducted in Southeast Asian countries, namely, Brunei, Cambodia, East Timor/Timor-Leste, Indonesia, Laos, Malaysia, Myanmar, Philippines, Singapore, Thailand, and Vietnam. Populations of non-human animals including livestock (cattle, buffalo, pigs, goats, sheep), companion animals (dogs, cats), peri-domestic/wildlife reservoirs (rats/rodents, bats), and other relevant species are included. Studies measuring the seroprevalence of *Leptospira* exposure as measured by PCR (in some species) and serology (primarily MAT, ELISA, and rapid antibody tests), including serogroup/serovar patterns when reported are included with a timeframe of 2000–2025 to capture contemporary epidemiology. Cross-sectional and surveillance studies, including abattoir, farm, shelter, and community-based sampling are also considered. Study exclusions include purely human studies, case reports without seroprevalence, and vaccine/immunity challenge experiments.

However, there are key limitations regarding diagnostic heterogeneity in comparing the studies mentioned. Studies in animal seroprevalence variably use MAT, ELISA, PCR, and culture. Even within MAT, panels (23–25+ serovars) and cut-offs (1:20–1:400) differ, producing method-dependent prevalence and shifting predominant serovar calls such that multi-serovar reactions and highest titer assignment can misrepresent true exposure profiles. These issues undermine comparability across surveys and over time. There is also sparse molecular/culture confirmation as PCR and culture are less common methodologies than MAT in prevalence studies. When performed, PCR/culture prevalence is typically lower (reflecting active infection/shedding) than MAT-based exposure estimates, limiting our understanding of current transmission dynamics and the proportion shedding at any time.

Sampling bias also arises from mixed designs—national cross sections, passive clinical surveillance, and abattoir and shelter convenience samples—leading to over representation of sick or high-risk subpopulations and reduced population representativeness. Passive surveillance (e.g., Thailand 2010–2015) likely inflates estimates compared with probability based cross sectional designs. Furthermore, most detailed animal data derive from Thailand, Vietnam, and Malaysia. Data are sparse or absent for Brunei, Singapore, Timor Leste, Myanmar, and large swathes of Cambodia, with limited breadth in Laos and the Philippines, hampering regional generalization of *Leptospira* infection among animal populations. Non-overlapping sampling periods (e.g., 2001 vs. 2010–2015 vs. 2021), potential seasonality, and evolving MAT panels/cut-off also limit longitudinal trend assessment and serovar distribution comparisons. Few studies co-sample humans, animals, and environments with molecular typing to resolve transmission pathways and source attribution; consequently, connections between animal reservoirs and human disease burden remain only partially elucidated.

Standardizing diagnostic reporting and pairing serology with molecular detection would substantially improve interpretability. Representative, stratified surveys with repeated measures are needed to estimate population prevalence and seasonality. Filling geographic and taxonomic gaps—especially in Brunei, Timor Leste, Myanmar, under sampled areas of Cambodia and Laos, and within rodent and peridomestic wildlife communities—should be prioritized. Integrated One Health studies that co-sample humans, animals, and environmental matrices with genotyping would enable source attribution and inform control strategies tailored to local maintenance hosts and serovar ecology.

Animal reservoirs play a critical role in the maintenance and transmission of *Leptospira*. Domestic animals, particularly cattle and swine, are the most important reservoir hosts that contribute to the varied regional epidemiology of leptospirosis [34]. *Leptospira* infection in large ruminants has been widely studied due to its economic impact resulting from reproductive failures such as infertility, premature births, and bovine fetal death as well as low milk production in dairy cattle [38,39]. In addition, pathogenic strains *L. borgpetersenii* and *L. kirschneri* have been detected in ruminant farms in the Philippines, Puerto Rico, and the USA that includes cattle and water buffaloes, suggesting that bovine leptospirosis can potentially cause economic losses in the livestock industry [40,41]. In addition, serovars Hardjo, Australis, and Shermani have been reported in bovine populations in nearby countries such as India, revealing a complex serovar distribution highly influenced by environmental and husbandry practices [25,32].

Global seropositivity for pigs (*Sus scrofa*) has been estimated to be at 21.95%, with high estimates coming from South America, North America, and Africa. Serogroups Icterohaemorrhagiae, Pomona, and Autumnalis dominate the infecting strains, which could pose a risk for human transmission especially those working in large-scale pig farms and backyard farms [42]. Pigs have also been studied to sustain *Leptospira* transmission in the environment by way of their behavior such as foraging or direct contact of their snout with the soil, wallowing in the mud, and contaminating the waterways through runoff sewage from pig farms [43,44]. This behavior poses a cross-infection with other transmissible bacterial infectious diseases as these animals are confined in tight spaces and interaction with wild animals is a likely possibility. However, research on the environmental transmission of the pathogenic *Leptospira* in pigs remains limited as most studies focuses on the capacity of wild pigs as a reservoir host and the transmission of the disease across swine and livestock [43,45].

Dogs, on the other hand, are maintenance hosts of several pathogenic *Leptospira* species mainly *L. interrogans* serovar Canicola, Icterohaemorrhagiae, Grippotyphosa, and Pomona [41,46,47]. However, their proximity to humans does not result in leptospirosis outbreaks but rather contributes to environmental contamination. Dogs have been known to excrete 1.6 × 10^5^ leptospires in their urine every day and are able to spread across great distances largely due to their movement through human-mediated activities [48]. Available data from other countries in the Southeast Asian region indicate similarly wide ranges of seroprevalence in canine populations, which includes 56.5% in the Philippines, 28.5% in Vietnam, and 22.5% in Malaysia, suggesting that even within the same region there exists marked heterogeneity in exposure levels to *Leptospira* spp. [34]. In addition, urinary shedding of *Leptospira* DNA, which is a marker of active infection, was found in 4.4% of the animals, further demonstrating that beyond past exposure, a subset of dogs maintains an active infection and may serve as a source of environmental contamination [46,49].

Stray and shelter dogs, predisposed to increased environmental exposure and contact with rodent reservoirs, tend to exhibit higher seroprevalence when compared to well-managed working dogs [36,37]. Working dogs, regularly maintained by government canine units and often subject to routine vaccination protocols, have shown low seropositivity (3.1%), even though these dogs were annually vaccinated using bivalent or quadrivalent vaccines [37]. Conversely, shelter dogs and dogs presenting with clinical conditions such as kidney and/or liver disease demonstrate considerably higher rates recorded as high as 42.7% [36].

There is also widespread serologic evidence of infection of domestic cats by *Leptospira* spp. However, based on rare reports of clinical disease, cats are considered disease-resistant when compared with other animal species that is usually infected with *Leptospira* [50,51]. Pathogenic leptospires have been detected in the urine of up to 20% of apparently healthy cats using PCR and culture; thus, cats may act as reservoir hosts [52,53]. However, other studies worldwide have shown lower prevalence of leptospiruria (0% to 9.35%), even in free-roaming cats [54,55]. Cats may be an under-recognized source of pathogenic leptospires in some regions and should be considered in One Health investigations to explore the role of environmental transmission in free-roaming potential hosts. These investigations could be valuable in understanding disease transmission dynamics in the aspects of studying factors affecting urban and rural human settlements.

Rodents are widely recognized reservoirs of pathogenic *Leptospira* as they are significant in maintaining the pathogen cycle and facilitating spillover into human populations [56,57]. In Southeast Asia, seroprevalence rates greatly vary depending on species, geographical location, and the type of diagnostic methods used. Studies in rural settings in Malaysian Borneo including wet markets reported a seroprevalence of 32% among wild rodents. This represents a significant difference compared with earlier urban studies in Sarawak that reported a prevalence of 5.6% [29,58]. Rodent species are among the most successful urban dwellers that have successfully adapted to human settlements. Increasing urbanization allowed the increase of these rodent populations in urban and rural environments, which can be both an economic and health concern. Wild rats (*Rattus* spp.) including the Norway/brown rat (*R. norvegicus*) and the black rat (*R. rattus*), as well as *R. rattus diardii*, *R. tiomanicus*, *R. exulans*, *R. agriventer*, *Maxomys whiteheadi*, and *Sundamys muelleri* have all been identified as important *Leptospira* maintenance hosts as their global distribution drives human leptospirosis infections [59].

Tropical and subtropical countries, including Southeast Asia, have been associated with rodent infestations due to the rapid growth of urban areas [58]. Along with the urbanization of spaces comes the increase in informal architecture as well as the worsening of sanitation conditions that offer favorable environments for these species to proliferate [60]. Recent studies involving the surveillance and determination of the genetic diversity of rodent populations have been conducted to trace the transmission of *Leptospira* in urban cities, including increasingly urbanized areas, as these areas are rapidly altering the structure and landscape of the environment enabling the increase in the incidences of human-pathogen contact [61,62,63]. Assessment of the prevalence of the pathogen is an important component of epidemiological surveillance and habitat management for curbing the possibility of disease outbreaks.

Other countries in and neighboring Southeast Asia also exhibit high seroprevalence among wild animal populations. In the Philippines, leptospiral infection among wild animals has been reported, with one study noting seropositivity rates as high as 80% in a sample of wild animals, emphasizing intense exposure in natural habitats [41,64]. Comparative surveys in Sri Lanka have also identified significant leptospiral carriage among peridomestic rodents and livestock, further supporting the hypothesis that wild and domestic animals contribute substantially to the region’s overall disease burden [65]. Despite these high figures, a comprehensive accounting of seroprevalence across all wild animal species is complicated by the relatively sparse sampling of species such as reptiles, squirrels, shrews, bats, and birds, which in some studies have shown much lower seropositivity. One meta-analysis found seroprevalences of 14.0% in reptiles, 5.0% in squirrels, 0.8% in shrews, 0.7% in bats, and 0.6% in birds [34]. This disparity highlights the fact that while rodents generally serve as robust reservoirs in terms of both infection intensity and transmission potential, other wild animal species might be less significant in sustaining transmission cycles or may simply be under-represented in the existing literature.

The interplay between animal and human seroprevalence is an important aspect in pathogen surveillance to develop effective management strategies to mitigate the serious effects of the disease. Livestock infected with pathogenic *Leptospira* serovars can experience reproductive and production losses, while asymptomatic infections in companion animals may facilitate the silent maintenance of the bacterium within communities [25,59]. Furthermore, the presence of multiple serovars in a single geographical area or even within a single animal complicates efforts at prevention and control because available vaccines typically cover only a limited number of serovars and may not offer cross-protection against emerging local strains [66]. The evident overlap between the serovar profiles found in domestic animals and those identified in human infections strongly suggests that transmission cycles are intimately linked to close human–animal interactions in the region. This scenario is particularly concerning in rural and smallholder farm settings where the boundaries between human habitation and animal husbandry are often blurred. Consequently, public health strategies must incorporate robust veterinary surveillance as well as targeted interventions to reduce environmental contamination [67]. Integrated One Health approaches that simultaneously address animal vaccination, rodent control, water sanitation, and community education are likely to be the most effective means of reducing the overall burden of disease in the region [68]. Overall, seroprevalence in Southeast Asia remains high and heterogeneous, reflecting both true epidemiologic variation and methodological inconsistencies.

## 3. Effects of Geographic and Climatic Influences, Urbanization, and Climate Change on Leptospirosis Transmission

Southeast Asia’s tropical climate, with high ambient temperatures, abundant rainfall, and extended wet seasons, creates conditions conducive to the survival and transmission of *Leptospira* in the environment (Table 3) [10,69]. Heavy rainfall and flooding events, which are common during the monsoon seasons, contribute to the dispersal of the pathogen through water and soil, enhancing human and animal exposures. Flood events not only increase the volume of contaminated water but also compromise sanitation and increase the interface between human populations and animal reservoirs, particularly in urban slums and rural agricultural settings. The magnitude of these risks is also exacerbated by regional climatic factors, urbanization, population growth, and the country’s socioeconomic status. Studies of outbreaks from Southeast Asian countries such as Thailand, Lao PDR, and Cambodia have shown an uptick in cases of leptospirosis in recent years [10]. Most of the infections not only come from urban areas but also from wetlands, rice fields and forestry work, which suggests that *Leptospira* infection is also driven by habitat landscape and not just rodent species [57].

Climatic factors have been observed to contribute to the spread of leptospirosis [85,86]. The amount of rainfall has an indirect association with the increase of rodent population in the area since increased rainfall can cool down environments, allowing rodents to thrive and reproduce [87]. Tropical environments characterized by monsoonal rainfall not only provide the moisture required for *Leptospira* survival but also promote the accumulation of contaminated water in the environment. In Southeast Asia, intense monsoon seasons generate flooding that disperses contaminated soil and water, directly linking rainfall events with subsequent disease outbreaks. For instance, studies conducted in the Philippines and Thailand demonstrate that leptospirosis incidence is positively correlated with heavy precipitation and flooding events, with outbreaks commonly following major rain events with lags ranging from one to several weeks [12,15]. These conditions enable the leptospires to survive longer in environments as periodic rains constantly keep the soil waterlogged further, establishing the evidence that environmental soil is a reservoir for *Leptospira* [88]. Furthermore, pathogenic *Leptospira* strains were also found to be able to survive even in seawater for 4 days post-storm surge, which indicates that disease outbreak may occur in disaster-stricken areas where seawater inundates the land during storm surges [89].

The recurrent seasonal pattern observed in leptospirosis incidence across Southeast Asia also highlights the importance of climatic seasonality. In regions such as Thailand, the rainy months between May and October witness a significant increase in case numbers, which is attributed to the seasonal intensification of rainfall and consequent waterlogging [12,14]. In other parts of the region, similar trends have been observed where periodic climatic variations modulate soil moisture and temperature, thereby directly influencing leptospiral survival and propagation [9,16]. The seasonality not only correlates with natural climatic cycles but also coincides with agricultural calendars, further compounding human exposure through increased contact in flooded fields and natural water bodies [72].

Temperature is another key climatic parameter influencing leptospirosis prevalence. The region is characterized by warm average temperatures that create optimal conditions for the bacterial survival of *Leptospira*. In many Southeast Asian countries, ambient temperatures consistently in the range of 27 to 28 °C have been associated with a higher number of leptospirosis cases [12,14]. Warm conditions also support recreational and occupational activities in water, further exacerbating exposure risks. Despite the ubiquity of these warm conditions, studies suggest that variations in temperature, particularly in endemic regions, are less significant than changes in rainfall or soil moisture [9,90]. High humidity (70–90%) combined with warm temperatures create environmental conditions that favor extended bacterial survival thus provides a supportive microenvironment for the persistence of pathogenic *Leptospira* in natural water and soil [71]. However, while temperature establishes a baseline for bacterial activity, it is the episodic heavy rains and subsequent increases in moisture that have a direct impact on case incidence [65,72,91]. These climatic features render Southeast Asian countries particularly vulnerable, as their equatorial location imposes both high temperatures and frequent cycles of humidity driven by monsoonal rains.

Land use plays a pivotal role in shaping the epidemiology of leptospirosis. Agricultural areas, particularly those used for rice cultivation, create conditions that are highly conducive to rodent infestations and the maintenance of waterlogged fields for prolonged periods [76,77]. In Southeast Asia, rice paddies are prevalent and are known to produce environmental conditions compatible with the bacterial survival of *Leptospira* [9,69]. Numerous studies have shown that wet fields during the rainy season significantly correlate with increased leptospirosis cases due to frequent human–water contact during agricultural activities [73,75]. Moreover, regions characterized by primary rice crop arable areas demonstrate a positive association with leptospirosis incidence, while agriculturally intensive sites may experience a more complex relationship with disease prevalence due to effective water management practices reducing exposure risk [74]. The presence of paddy fields also contributes to creating a microenvironment with persistent moisture, which supports the persistence of the bacteria, and serves as a habitat subdivision that favors rodent reservoirs [73].

Vegetation and land cover characteristics are critical to understanding the environmental reservoirs of leptospirosis. Dense vegetation, with high values of indices such as NDVI (normalized difference vegetation index), may provide favorable microclimatic conditions that support rodent populations and *Leptospira* survival [81]. Forested areas, degraded shrub lands, and rice fields characterized by robust vegetation coverage have been associated with higher leptospiral carriage in rodents [78]. In several studies, the presence of forested enclaves and natural plantations, including rubber plantations, has emerged as a risk factor due to increased natural water retention and elevated humidity in these microhabitats [57,80]. Notably, the land cover types in Southeast Asia, including both cultivated and natural landscapes, contribute substantially to the geographic heterogeneity observed in leptospirosis outbreaks.

Urbanization of habitats is a widespread and significant process that modifies the existing landscape extensively and often permanently. These changes are often marked by a reduction in biodiversity due to the loss of habitat for endemic species and allow the proliferation of wildlife in highly urbanized areas where there are adequate resources [17,79]. A high prevalence of infection has been observed in urban slums and low-lying areas due to poor sanitation, informal infrastructures, frequent contact with floodwater, and poor pest control [92,93]. However, urbanization alone is not a significant driver of leptospirosis since a high prevalence of the disease in rural areas has also been observed, especially in agricultural workers and those who interact with domestic animals including wildlife [48,78,94]. It is also worth noting that socioeconomically disadvantaged communities in both urban and rural areas have disproportionate rates of leptospirosis [82]. Additionally, this observed disproportionate rates can be heightened during natural disasters such as typhoons, which cause flooding due to lack of resources, delays in government assistance, and unequal access to medical care [83,84]. These factors have been especially amplified in low- to middle-income countries where natural disasters are a common occurrence. Tropical regions such as Southeast Asia have seen a higher prevalence of leptospirosis due to favorable environmental conditions that encourage the proliferation of zoonotic pathogens [9].

**Figure 1:**
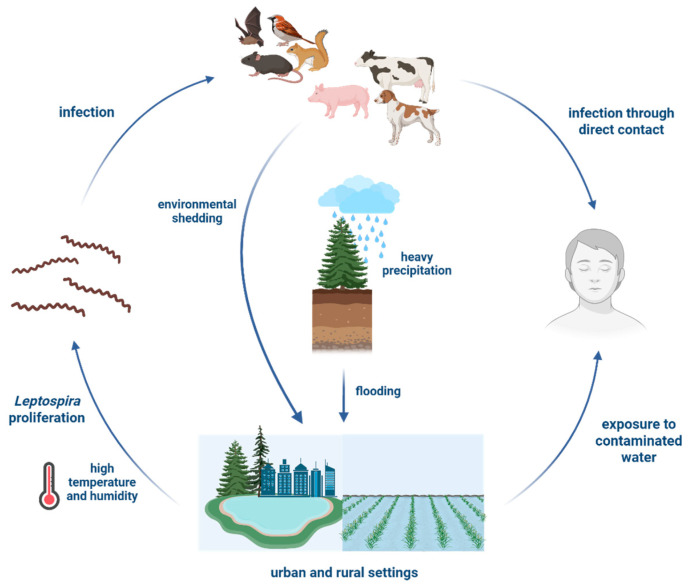
Environmental transmission of *Leptospira* sp. and climatic factors influencing its cycling. Various factors including temperature, climatic conditions, and urban and rural settings contribute to the proliferation of *Leptospira* in the environment.

Numerous studies consistently show that climatic factors are fundamental in driving leptospirosis transmission in Southeast Asia. Figure 1 illustrates how environmental contamination is sustained by a combination of climatic and anthropogenic factors revealing the complexity of *Leptospira* transmission. Despite this consensus, substantial uncertainties remain regarding the mechanistic pathways and relative contributions of various climatic variables. Although rainfall and flooding are consistently observed to affect leptospirosis transmission, the precise non-linear relationships, lagged effects, and interactions with temperature and humidity are not yet fully understood [78]. The use of rainfall as a proxy for complex ecological drivers, such as rodent population dynamics and environmental wetness, introduces additional uncertainty. Moreover, heterogeneity in study design, differences in exposure definitions, and the variability in diagnostic criteria across studies further complicate a clear quantification of the climatic impacts on leptospirosis transmission [78,95].

Finally, while urban settings may experience distinct transmission pathways, often characterized by different reservoir hosts and environmental management challenges, rural areas commonly face more direct exposure to floodwaters and agricultural practices. This geographical and socio-economic diversity leads to uncertainties in how climatic drivers operate in different settings, complicating the development of universally applicable predictive models [95,96]. The implications for public health and disease management in Southeast Asia are significant. Integrating high-resolution, local meteorological data into spatial-temporal risk models can improve early warning systems and guide targeted interventions. Such models have the potential to forecast outbreaks based on parameters like cumulative rainfall, flood events, and temperature anomalies, thereby enabling public health authorities to allocate resources more effectively and implement timely preventive measures, such as enhanced rodent control and appropriate water sanitation strategies [72,97].

## 4. Spatial Epidemiological Approaches in Tracking the Environmental Transmission of Leptospirosis

The environmental dependency, the geography and climate of Southeast Asia play central roles in driving the incidence and dynamics of leptospirosis outbreaks, is thus an important feature in the persistence of the pathogen [78,93]. This coupled with underreporting and misdiagnosis in both rural and urban settings, necessitates the need to utilize spatial epidemiological methods that can integrate diverse datasets to better understand transmission patterns and risk factors [98].

In recent years, geographic information systems (GIS), remote sensing (RS), and advanced statistical modeling have been used as tools for visualizing, exploring, and predicting leptospirosis risk at multiple geographical scales that can help improve surveillance and provide framework for public health interventions [99]. These spatial epidemiological approaches are particularly valuable in resource-limited and endemic regions where traditional surveillance systems may fall short as they facilitate rapid identification of high-risk areas enabling timely and evidence-based interventions. GIS is central to the study of the spatial epidemiology of leptospirosis. It enables the mapping and visualization of case distribution, environmental risk factors, and socio-demographic factors across various spatial scales [100]. This allows researchers to create maps that graphically represent the prevalence and incidence of leptospirosis which reveals its heterogenous pattern and potential high-risk hotspots where targeted interventions such as rodent control or improvement of sanitation may yield substantial benefits [101]. Risk mapping techniques have also been applied to the epidemiological modeling that quantified the relationship between disease incidence and environmental and socio-economic factors [102,103]. The resulting risk maps serve as a tool for facilitating targeted public health responses by highlighting regions where interventions might have the greatest impact. Regression models using Bayesian geostatistical approaches, on the other hand, enables the integration of prior observed information to produce posterior distributions for risk estimates [104,105]. This, coupled with the application of geographically weighted regression, allows for the local modeling of the effects of various risk factors that captures the shifting mechanics of disease transmission, which ultimately leads to more nuanced risk mapping and intervention strategies [106].

Emerging machine learning techniques have also been increasingly integrated with spatial epidemiological analyses to enhance the predictive accuracy of leptospirosis risk maps. For instance, predictive risk mapping studies have demonstrated that integrating predictors such as rainfall, temperature, and vegetation indices into machine learning algorithms could produce highly accurate maps that are able to forecast potential outbreaks in a given region under different climate conditions [107,108]. These data-driven practices have also been utilized as early warning systems for preventive interventions prior to the anticipated increase in leptospirosis incidence particularly in periods of heavy rainfall and flooding. Remote sensing captures the environmental determinants of leptospirosis risk as it generates spatially continuous data that are crucial for understanding the ecological drivers of transmission [109]. Satellite sensors provide vital information on land cover, vegetation and water indices, temperature, and precipitation patterns all of which are known to heavily influence the survival and dispersal of *Leptospira* in the environment. Spatio-temporal modeling has also been utilized for understanding the seasonality and lagged effects of climate factors on disease outcomes [110]. Several models have demonstrated that leptospirosis incidence can be significantly correlated with rainfall intensity and flooding with lag periods ranging from a few days to several weeks [111,112,113]. These techniques have been instrumental in providing valuable insight into disease dynamics in rapidly changing environmental conditions. Such approaches thus enable the identification of seasonal peaks and the emergence of transient hotspots that can inform not only localized interventions, but also broader region-specific strategies tailored to the specific temporal dynamics of the disease [85,114].

The application of these new technologies in tracking disease transmission across the region introduces additional layers of complexity and opportunities for researchers to capture fine-scale environmental determinants that are otherwise difficult to measure on the ground [115]. Thailand’s flood risk modeling framework has been structured around a systematic risk management approach that emphasizes data collection, risk identification, assessment, and treatment, which is pivotal for mitigating waterborne hazards and associated disease outbreaks such as leptospirosis. Recent improvements have made possible to project urban flood risk areas in the coming years using a combination of various climatic and geographic factors highlighting the need for improved floodwater drainage systems and resilient urban planning [116,117,118]. In parallel, Indonesia’s GIS surveillance strategy leverages high-resolution spatial data to identify leptospirosis hotspots and associated environmental risk factors. The integration of advanced machine learning techniques with remote sensing has proven instrumental in predicting outbreak patterns and enabling targeted public health interventions [119,120]. Both approaches underscore the critical role of integrating comprehensive spatial analysis with structured risk management. The Thailand flood risk framework offers a robust methodology for planning and managing flood-related risks, while Indonesia’s GIS-based surveillance system effectively delineates disease risk dynamics for informed intervention strategies.

However, the practical implementation of spatial epidemiological approaches requires careful consideration when it comes to scalability and transferability across various geographical settings. For instance, studies conducted in densely populated urban centers often reveal different risk factors compared to those observed in rural or peri-urban settings which highlights the importance of context-specific model calibration and validation [78,103]. Inter-country variability also plays a crucial role as differences in healthcare infrastructure, diagnostic capacity, and surveillance systems between Southeast Asian countries lead to inconsistencies in data collection and reporting [99,112]. Consequently, these disparities introduce heterogeneity into spatial models and necessitate conservative methodological approaches to avoid overestimating or underestimating disease burden. This more nuanced understanding is important in implementing public health interventions and strategies that are cost-effective and responsive to local epidemiological settings. Furthermore, integration of animal surveillance data with human case reports represents a critical component in upgrading spatial epidemiology for a holistic One Health approach. Studies that combine spatial analyses of rodent populations, livestock infestations, and canine seropositivity could provide a clearer picture of the transmission dynamics and potential spillover effects that may precede human outbreaks [121,122,123]. Such integrative approaches could not only allow for more precise forecasts but also facilitate the improvement of multidisciplinary intervention strategies that could address the complex interplay between humans, animals, and the environment.

While substantial progress has been made in integrating geospatial modeling in leptospirosis surveillance, future research must also address the persistent challenges in data heterogeneity from poor surveillance data especially in resource-limited settings, limited access to high-quality geocoded epidemiological data, and the lack of technical expertise required to implement complex spatial models [99,101,124]. The financial burden associated with establishing, maintaining, and improving quality surveillance and diagnostic facilities is also substantial. This resource intensity limits the availability of representative data and curtails the ability to perform comprehensive laboratory confirmatory tests, which are critical to reliable spatial analyses [112,124]. Therefore, collaborative initiatives that bring together experts in epidemiology, remote sensing, data science, and public health policy are essential to overcome operational barriers to ensure that these methods are scientifically robust and applicable to various geographical settings. The integration of environmental, epidemiological, and socioeconomic data underlines the complexity of leptospirosis transmission and highlights the need for continuous innovation in spatial analytic methods to keep up with the emerging challenges brought about by urbanization, climate change, and changing population dynamics.

## 5. Leptospirosis Diagnostics in Humans and Implications in Public Health Disease Management Strategies

Current diagnostics for leptospirosis are characterized by a multifaceted and evolving landscape that integrates traditional serological methods and culture-based approaches with advanced molecular techniques and rapid point-of-care assays (Table 4). Clinical signs of leptospirosis such as fever, headache, myalgia, and jaundice often mimic those of other tropical infections like dengue or influenza; thus, laboratory confirmation is indispensable for an accurate diagnosis [125]. Traditionally, diagnosis has relied on serologic assays and culture, both of which are limited by delays inherent to antibody production and the prolonged incubation periods required, respectively [126]. In recent years, molecular diagnostic techniques, including various PCR-based approaches, have markedly improved early detection, thereby facilitating prompt initiation of antimicrobial therapy.

### 5.1. Traditional Diagnostic Methods

Culture methods using specialized media such as Ellinghausen–McCullough–Johnson–Harris (EMJH) remain the definitive diagnostic technique since they allow for the isolation of live *Leptospira* organisms [127]. However, these methods are generally impractical for routine clinical use due to long incubation times as culture may take weeks to months requiring the need for rigorous biosafety measures. Serologic assays remain a mainstay in the diagnosis of leptospirosis and are widely used in public health surveillance to provide epidemiological insights [128]. The microscopic agglutination test (MAT) is considered the gold standard for serological testing, as it allows for the detection of specific antibodies and the identification of the infecting serogroup [128,129]. However, despite its specificity, its reliance on live leptospiral cultures, the need for skilled personnel, and the requirement for paired serum samples collected several weeks apart limit its practical application in clinical settings.

Alternative serologic methods, such as IgM ELISA, offer faster turnaround and ease of use by detecting antibodies from around 5–7 days after symptom onset, yet these too suffer from cross-reactivity and may not reliably distinguish between current and past infections [130]. Indirect immunofluorescence assays similarly provide rapid and sensitive results, although they generally lack the ability to determine the specific infecting serovar. Tests such as MAT and ELISA are inherently limited by the delayed antibody response, with IgM antibodies typically detectable only after 5–7 days from symptom onset; this delay often precludes early diagnosis and can result in missed opportunities for timely intervention [131]. As a result, serologic assays are typically used in conjunction with molecular tests, particularly for confirmatory diagnoses following an initial negative result in the acute phase. While traditional methods have significantly contributed to leptospirosis diagnosis and epidemiology, their limitations in sensitivity, speed, and operational complexity highlight the need for alternative approaches.

Methodological heterogeneity, however, is a pervasive challenge in the diagnostic evaluation of leptospirosis. Central to this issue are the differences in the reference standards, particularly the use of MAT versus ELISA. MAT is widely regarded as the serologic gold standard; however, its diagnostic performance is highly sensitive to the choice of cut-off thresholds. In many studies, a single acute-phase MAT sample is employed, whereas others require a 4-fold rise or seroconversion with paired samples. This variability can lead to both false-positive and false-negative results due to persistent IgM antibodies and differences in antigen panel composition [132,133]. ELISA, used extensively for screening, also suffers from marked heterogeneity because different studies employ various cut-off criteria. Some investigations optimize ELISA thresholds to reflect population-specific immune responses, while others adhere strictly to manufacturer instructions. This results in significant discrepancies in reported sensitivity and specificity, undermining the comparability of findings across studies [134,135]. Consequently, the lack of a consistent, universally accepted cut-off hampers efforts to standardize diagnostic protocols and complicates the integration of ELISA results into clinical decision-making [134].

In addition to assay-specific variability, differences in study design further contribute to heterogeneity. Many studies suffer from small sample sizes, which can produce unstable estimates and limit the external validity of diagnostic accuracy measures. Smaller cohorts are more susceptible to statistical imprecision, while larger, more robust studies tend to yield more reliable diagnostic performance metrics [132]. Variability in sample size across studies complicates meta-analyses and systematic reviews by increasing between-study variability and reducing the overall confidence in pooled estimates. Selection bias is another critical factor contributing to methodological heterogeneity. Studies employing retrospective or two-gate case–control designs often enroll control groups that may not be representative of the target population. For instance, the use of healthy donors or patients with clinically similar illnesses such as dengue may result in artificially inflated specificity and sensitivity estimates. In contrast, prospective designs with consecutive patient enrollment provide more reliable data by minimizing selection bias and enhancing the generalizability of findings [134].

Timing of sample collection relative to the onset of symptoms (days post-onset, DPO) further exacerbates the variability. Sensitivity of both MAT and ELISA is known to improve markedly after the first week of illness, yet many studies do not carefully control or report the DPO of collected samples. This lack of standardization in sample timing contributes to inconsistent diagnostic accuracy estimates and complicates comparisons across diverse study settings [133]. Addressing these issues requires the adoption of standardized diagnostic criteria, robust prospective study designs, and careful control of sample timing to enhance comparability and improve the clinical utility of leptospirosis diagnostics [132].

### 5.2. Molecular Diagnostic Approaches

Molecular diagnostics have transformed leptospirosis detection by enabling the identification and quantification of leptospiral DNA directly from clinical specimens. Real-time PCR (qPCR) assays, for example, target specific gene regions such as lipL32, a gene present in pathogenic and clinically relevant *Leptospira* strains, and have thus demonstrated rapid detection capabilities earlier in the disease course than serologic methods can achieve [136,137]. The quantitative aspect of qPCR not only aids in early diagnosis but also facilitates the assessment of bacterial load, which can be correlated with disease severity and prognosis. Multiplex PCR assays have further been developed to simultaneously detect and differentiate between various leptospiral serogroups that may be missed by conventional assays. Although PCR-based techniques offer improved sensitivity during the leptospiremic phase, typically within the first week of illness, their performance is highly dependent on the quality of the extracted nucleic acids and can be compromised by inhibitors present in blood or urine samples. Additionally, factors such as prior antibiotic therapy may reduce detectable bacterial DNA. Newer isothermal amplification techniques such as recombinase polymerase amplification (RPA) and loop-mediated isothermal amplification (LAMP) are being explored as cost-effective and rapid alternatives that eliminate the need for thermal cycling equipment, rendering them especially promising for point-of-care applications in resource-limited settings [138,139].

These advances illustrate how modern molecular diagnostics are reshaping early detection strategies for leptospirosis, enabling clinicians to initiate treatment during the critical early phase of infection. However, the narrow window of detectable leptospiral DNA in blood samples, typically confined to the first week of illness, and the possibility of reduced sensitivity after antibiotic initiation remain critical issues. Additionally, the performance of PCR and related assays is contingent upon high-quality nucleic acid extraction since the presence of inhibitors in clinical samples can compromise assay results, leading to false negatives.

### 5.3. Rapid Diagnostic Tests and Point-of-Care Assays

Rapid diagnostic tests (RDTs) and point-of-care assays are designed to overcome infrastructural constraints by providing quick results with minimal technical requirements therefore can be performed by non-laboratory personnel such as clinicians [134,135]. Immunochromatographic lateral flow assays that detect anti-leptospiral IgM antibodies can yield results in as little as 20 min and require no sophisticated laboratory equipment, making them particularly attractive for use in primary care settings and remote areas. However, the performance of these tests is highly variable; sensitivity has been reported to range from as low as 79% to as high as 93.9%, while specificity may range from 52.3% to 100% depending on the test and the geographical region, attributable in part to variability in regional serovar prevalence [140,141].

Other rapid assays, such as dipstick tests and flow cytometry-based methods, are under active investigation to enhance diagnostic accuracy and performance under field deployment, but further validation and optimization are necessary before they can be widely implemented [142]. Rapid diagnostic tests, although useful for quick screening, exhibit wide variability in sensitivity and specificity across different populations and regions, highlighting the influence of local serovar diversity and prior exposure. Despite their limitations, rapid diagnostic tests remain a critical component of diagnostic algorithms in resource-poor settings, particularly when used as a preliminary screening tool that is subsequently confirmed by more definitive methods, such as PCR or MAT.

### 5.4. Combined Diagnostic Approaches and Integrated Algorithms

**Figure 2:**
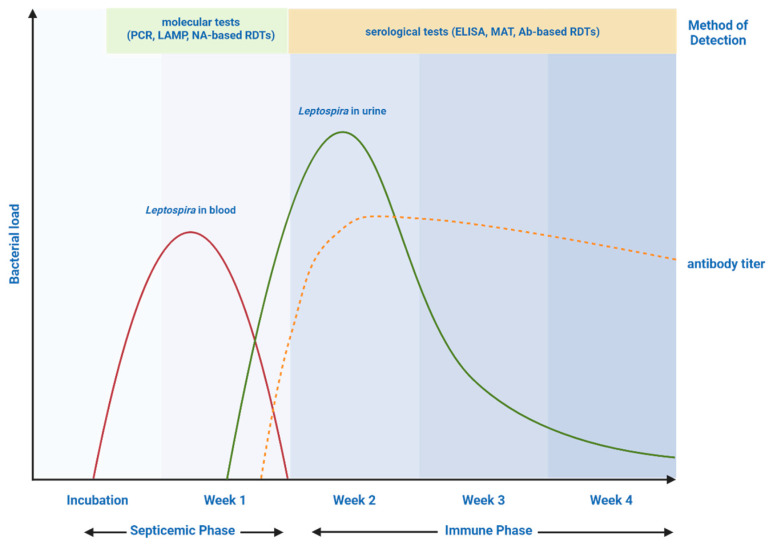
Course of leptospirosis infection and the corresponding diagnostic tools to use for detection. Molecular tests (PCR, LAMP, NA-based RDTs), which are best used during the septicemic phase as the pathogen is detectable in the blood, work in tandem with serological tests (ELISA, MAT, Ab-based RDTs) that can detect the immune phase of the infection.

Because no single diagnostic method is capable of providing perfect sensitivity and specificity throughout all stages of leptospirosis, integrated diagnostic algorithms that combine multiple methods have become increasingly important. A common strategy involves using molecular techniques such as PCR within the first week of infection to capture cases during the early leptospiremic phase, followed by serological testing using MAT or ELISA during the immune phase when antibodies have developed sufficiently to confirm infection [143]. This combined approach leverages the strengths of each method. Specifically, molecular assays offer rapid and sensitive detection in the acute phase, while serologic tests provide confirmatory evidence and epidemiological insights (Figure 2).

Several clinical studies have demonstrated that implementing a dual testing strategy can improve overall diagnostic yield and facilitate earlier treatment interventions, which is particularly important given that the efficacy of antibiotics decreases as the disease progresses [126,144]. Moreover, the cost and technical complexity of advanced molecular diagnostics, including real-time PCR platforms, pose significant hurdles for implementation in low-resource settings where leptospirosis is most prevalent. These operational and technical challenges call for the need for continued research and validation to optimize existing assays and develop new diagnostics that are both accurate and accessible.

### 5.5. Future Perspectives and Recent Technologies

To address the current limitations, several promising avenues of research are under development. One major focus is on isothermal amplification methods such as recombinase polymerase amplification (RPA) and loop-mediated isothermal amplification (LAMP), which can yield rapid results without the need for complex thermocycling instruments, thereby reducing cost and increasing field applicability [138,145]. Preliminary studies have shown that RPA can achieve sensitivities and specificities comparable to qPCR while delivering results in as little as 35 min, making it a strong candidate for point-of-care testing in endemic regions. In addition, novel biomarker assays such as the detection of leptospiral sphingomyelinase in urine, are being explored for their potential to serve as adjuncts to PCR and serologic methods, potentially increasing diagnostic sensitivity during the later stages of infection [146]. Advances in biosensor technology and microfluidic platforms are also encouraging, with the prospect of developing integrated, portable diagnostic devices that combine rapid molecular amplification with real-time detection, thereby enabling decentralized testing with minimal infrastructure.

Some of the DNA biosensors that were developed used gold particle–carbon nanofiber composites, gold nanoparticle electrodes linked to graphene quantum dots, and tapered optical fibers that are highly specific to the *Loa22, lipL32*, and *secY* genes of the *Leptospira* spp. These biosensors prove to be sensitive with low limit of detections (LOD) ranging from 0.0077 to 12 ng/µL [147,148]. These DNA-based biosensors can be applied to develop a point-of-care test (POCT) for the rapid diagnosis of leptospirosis. However, these biosensors detect leptospiral DNA and thus require isolating the DNA from the samples prior to performing the test, which does not necessarily simplify the process.

In recent years, aptamers, which are stable, single-stranded nucleotides, have been gaining interest as a potential biorecognition element for pathogen and small molecule detection due to their high affinity for target molecules akin to that of antibodies [149]. Cell-specific aptamers have been developed to detect the *Leptospira* cell [150], which eliminates the need for DNA isolation. These aptamers target the LipL32 protein and were able to detect 57-119 CFU/mL of the pathogenic leptospires in environment and water samples [149,151,152]. This technology has the potential to be applied to point-of-care settings where early detection and surveillance of leptospirosis is of its utmost importance due to their improved sensitivity and specificity, a broader range of targets, lower production costs, and longer shelf life. Electrochemical biosensors also have been utilized to detect *Leptospira* species which offer a rapid response, higher sensitivity, simplicity, and ease of use compared to the traditional MAT. Microfluidic devices have also been developed to facilitate inexpensive, rapid, and easy detection of infectious diseases [153]. These tests highlight the potential approach for noninvasive leptospirosis diagnosis which addressed the critical need in clinical and public health settings.

Concurrent research into host-response profiling using transcriptomics, proteomics, and metabolomics may yield novel diagnostic markers that complement pathogen detection, offering a more comprehensive picture of disease status that could guide both diagnosis and treatment [154,155]. Furthermore, the integration of machine learning and artificial intelligence to analyze complex datasets derived from multi-modal diagnostic platforms is emerging as a promising strategy to improve diagnostic accuracy and predict clinical outcomes [110]. Together, these innovations signal a future in which leptospirosis diagnostics will be more rapid, sensitive, and accessible—capable of meeting the diverse needs of both high-resource and resource-limited settings.

**Table 4:** Current diagnostic methods in detecting leptospirosis.

Type of Test	Method of Detection	Advantages	Disadvantages	References
Culture technique	Isolation of live leptospires	- High specificity - Isolates can be characterized	- Long incubation time - Requires rigorous biosafety measures and training	[89,127,156]
Microscopic agglutination test (MAT)	Microscopic observation of antigen–antibody interaction	- Serovar specific - Widely accepted in commercial settings - Clinically validated	- Labor intensive - Longer turn-around time - Less sensitive during the early acute phase - Prone to misdiagnosis	[128,129,131]
ELISA using IgG or IgM antibodies	Spectrophotometric detection of antigen-antibody interaction	- Faster turnaround and high throughput - High reproducibility	- High potential for cross-reactivity - Cannot discriminate between current and past infection - Less sensitive during the early acute phase	[130,131]
Molecular techniques (conventional PCR, qPCR, multiplex PCR assay)	Detection of leptospiral DNA	- Rapid and real-time detection and quantification - More sensitive during the early acute phase - Differentiates between serogroups - Clinically validated	- Highly technical - Costly - Antibiotic therapy might give false negative results	[136,137]
Isothermal amplification	Nucleic acid amplification using a single temperature	- Rapid and cost-effective - Highly sensitive and specific - Does not require a thermal cycler - High potential for point-of-care	- Highly technical - Costly reagents - Not standardized	[138,139,145]
Rapid diagnostic tests (RDTs)	Detection of *Leptospira*-specific antigen or antibodies	- Rapid results - Minimal technical skill requirements - Does not require advanced equipment to produce result	- Varying specificity and sensitivity - Cannot identify infecting serovar - Require confirmatory testing (PCR, MAT, etc.) - Not standardized	[140,141,157]
Biosensor (DNA, electrochemical)	Biorecognition element (DNA probe, antigen, LPS, or whole cell)	- High sensitivity - Portability and real-time detection	- High cost of fabrication - Complex sample preparation - Sensitive to inhibitors - Not standardized	[147,148]
Aptamers	Stable, single-stranded nucleic acid (DNA or RNA) binding to target molecule	- Highly sensitive and specific - Cost-effective - Highly stable - High reproducibility - Flexibility in various detection platforms	- Highly technical and time-consuming - Sensitive to sample inhibitors - Not standardized	[149,150,151,152]

### 5.6. Implications in Disease Management and Impact on Public Health Policy

The evolution of diagnostic modalities is not only pivotal for individual patient care but also for strengthening public health surveillance systems. Rapid and accurate diagnosis facilitates timely antimicrobial therapy, reduces the risk of severe complications, and enhances outbreak control by enabling early detection of clusters of cases [158]. Moreover, the integration of molecular diagnostics into national surveillance programs has greatly improved the ability to monitor leptospiral strain diversity and geographic distribution, which are essential for developing targeted intervention and vaccination strategies [126]. In many regions, case definitions that combine clinical criteria with confirmatory laboratory tests are used to guide treatment and public health responses, which is particularly important in settings where leptospirosis is frequently underdiagnosed [143]. The ability to rapidly differentiate leptospirosis from other febrile illnesses using integrated diagnostic algorithms also contributes significantly to improved antibiotic stewardship and overall patient outcomes [159]. Moreover, diagnostic algorithms that incorporate both molecular and serologic testing can help distinguish between acute and past infections, providing a more accurate picture of disease burden and transmission dynamics essential for resource allocation and policy development.

Enhanced diagnostic capabilities not only improve individual patient outcomes but also have significant epidemiological ramifications. Accurate and timely diagnosis of leptospirosis is crucial for mapping disease incidence, monitoring transmission patterns, and implementing effective control measures, especially in endemic regions where the disease burden is high [160]. The adoption of advanced molecular diagnostics in surveillance programs facilitates real-time tracking of outbreaks and enables public health authorities to respond more rapidly and effectively. In addition, integration of data from these advanced diagnostic methods into global surveillance networks helps provide a more accurate estimation of leptospirosis incidence and informs the development of tailored prevention strategies [158]. The resulting data not only guide clinical interventions but also support policymaking and resource allocation for improving healthcare infrastructure in regions most affected by leptospirosis.

Furthermore, the translation of innovative diagnostic technologies from the research laboratory to routine clinical use requires close collaboration among multiple stakeholders, including academic researchers, healthcare providers, government agencies, and international organizations. Such collaborations are essential to standardize diagnostic protocols, ensure quality control, and build the necessary laboratory infrastructure in endemic regions [160]. Global health initiatives that promote knowledge sharing and capacity building are critical to overcoming the operational challenges that currently limit the widespread adoption of advanced diagnostic methods. Clinical trials that assess the impact of novel diagnostic approaches on patient outcomes and public health surveillance further underscore the need for integrated, multi-stakeholder efforts to enhance leptospirosis diagnosis.

## 6. Conclusions and Future Directions of Research

Leptospirosis remains a substantial health and socioeconomic burden across Southeast Asia, disproportionately affecting vulnerable populations in low- and middle-income countries. Its endemicity is largely shaped by environmental, occupational, and socioeconomic factors. Frequent contact between humans and infected animals—such as rodents, pigs, large ruminants, and companion animals—facilitates widespread environmental contamination with *Leptospira* bacteria, especially in communities with poor sanitation and limited access to clean water [96,161]. Urbanization, deforestation, and changing land use patterns increase human exposure to contaminated environments, while seasonal flooding and warm, humid climatic conditions further exacerbate the risk of outbreaks [115]. The seroprevalence of leptospirosis across Southeast Asian countries varies significantly due to inconsistent diagnostic practices and detection methodologies, making it difficult to accurately assess the true burden [160]. Nonetheless, consistently high infection rates among human and animal populations underscore the urgent need for comprehensive control strategies. Vulnerable groups—including agricultural workers, the urban poor, and those in flood-prone regions—are particularly at risk. A regionally coordinated health agenda that includes effective management, public health education, and strengthened health systems is crucial not only to reduce disease incidence but also to improve outcomes in populations affected by severe complications of the disease [162].

The disease’s broad clinical spectrum, ranging from mild febrile illness to severe complications, contributes to frequent misdiagnoses and delays in treatment. Clinical awareness, prompt management protocols, and early diagnosis are essential to mitigate the impact of severe complications of the disease [163]. Persistent gaps in surveillance and diagnostics significantly hinder the control of leptospirosis. Underdiagnosis is common due to its non-specific symptoms, limited clinical awareness, and diagnostic tools that are either too complex or unvalidated for widespread use in resource-poor settings [158]. Environmental modeling, which could aid in outbreak prediction, remains underdeveloped due to challenges in data integration, limited access to real-time environmental data, and the lack of full integration of spatial epidemiology approaches into national disease monitoring systems [99,112]. While early warning systems show promise, challenges remain in data integration and model validation as there is a need for robust, context-specific spatial epidemiological models. Addressing these gaps is essential for improving risk mapping, clinical management, and outbreak preparedness [99,101,124].

The development of effective vaccines against leptospirosis also remains an urgent priority to prevent severe disease outcomes. Understanding the pathogenesis and virulence factors of *Leptospira* species is significant for vaccine development and biomarker identification. The current landscape of leptospirosis vaccine research is characterized by a multi-pronged approach that integrates advanced antigen discovery methods, novel delivery systems, and potent adjuvants to overcome the inherent limitations of traditional bacterins [164]. Persistent challenges, including antigenic heterogeneity, the translation of animal model data to humans, and inconsistent protective efficacy, necessitate continued research. Future directions will likely focus on optimized multi-antigen formulations, prime-boost regimens, and improved immunization protocols to achieve broad-spectrum and long-lasting protection against leptospirosis [165].

A comprehensive One Health approach that fosters collaboration across multiple sectors and disciplines is critical for addressing leptospirosis in Southeast Asia. Policy recommendations include integrated human–animal–environment surveillance, standardization and validation of diagnostic tools, and targeted vaccination strategies to break transmission cycles [158]. Public education, occupational safety measures, and improved urban infrastructure, especially flood control and sanitation—are vital to reduce exposure risks [162]. Interdisciplinary collaboration and strong governance frameworks are essential for implementing these strategies effectively. Future regional efforts should prioritize scalable innovations in diagnostics and harmonized surveillance systems to ensure sustainable disease control and improved health outcomes across sectors.

## Figures and Tables

**Table 1:** Summary of the selected human seroprevalence studies on leptospirosis infection across Southeast Asia.

Country	Study Years/Setting	Sampling Population	Assay (Cut-Off)	Reported Seroprevalence	References
Cambodia	2007–2009 community febrile surveillance (Kampong Cham)	Febrile subjects < 20 years old; 2044 subjects, 2358 convalescent samples	IgM ELISA on convalescent; MAT confirmation on seroconverters (MAT ≥ 1:100 used for confirmation)	IgM convalescent positive: 630/2358 = 26.7%; seroconversions (acute negative → convalescent positive): 100 seroconversions among paired samples (15.8% of ELISA-positives); MAT-confirmed seroconversions: 17/2358 = 0.72% overall	[19]
Vietnam	November-December 2019 (North, northern Central, South Vietnam)	Healthy adult surveys (N = 600 blood samples)	MAT ≥ 1:100	9.5% in asymptomatic Vietnamese population	[20,21,22]
	Community surveys in southern Vietnam (March 2003)	Primary school aged children 7–12 years old (N = 961 blood samples)	ELISA-IgG and IgM	ELISA-IgG: 12.8% (ages 7–12 years); child cohort seroconversion ~10.4% over 3 years in one cohort; ELISA-IgM: 5.4%	
	Cross-sectional study in the Mekong Delta	Randomly selected participants aged 15–60 (N = 1400 blood samples)	Variable MAT cut-offs	~11.2–20.5% (overall reported ~18.8%)	
Lao PDR	Reported in regional synthesis/surveys (rural)	Rural adult communities (age 15–78 y/o)	MAT ≥ 1:100	~23.9% in rural samples	[23]
Thailand	Samples collected March–September 2020 (blood donors)	Healthy blood donors across 5 regions (N = 1053)	MAT (panel) and anti- IgG ELISA (commercial genus-level ELISA)	MAT: no evidence of recent infection among donors; IgG ELISA positives: 18/1053 = 1.7% (past exposure)	[24,25,26]
	2010–2015 (humans and livestock)	N = 1990 human serum samples	MAT ≥ 1:100	23.7%	
	Hospital-based (2001–2012; 2 periods of patient recruitment)	Febrile patients	IFA ≥ 1:100	40% (first period) 12.7% (second period)	
Indonesia	Surveys and flood-associated sampling (reported in regional review) from the early 2000s	Small community/outbreak sample sets (examples: N = 139 and N = 418)	MAT/serology described in review (method details variable across reports)	18.7% of 139 (survey), 12.0% of 418 (flood associated)	[16]
Philippines	January 2017–April 2018 (Metro Manila)	Cross-sectional (N = 105 employed sewer workers)	MAT ≥ 1:100	4.8% (5/105)	[27]
Malaysia	2018 (wet market workers in Kelantan)	232 respondents (cross-sectional study)	MAT ≥ 1:100	33.6%	[28]
Other SEA countries (Myanmar, Singapore, Brunei, Timor-Leste)	Data coverage variable; many country-level gaps noted in regional reviews	Few representative studies, comparable community serosurveys located in the retrieved set	Reporting inconsistent; occupational/small group studies	Representative, comparable, up-to-date seroprevalence estimates not found in the retrieved evidence for several countries	[29,30,31]

**Table 2:** Summary of selected animal seroprevalence studies on leptospirosis infection across Southeast Asia.

Country	Species	Representative Study (Years)	Sample Size (If Reported)	Assay (Cut-Off)	Reported Seroprevalence	Predominant Serogroups/Serovars	References
Thailand	Cattle	National cross-sectional survey	N = 9288 cattle	MAT; 24-serovar panel; cut-off ≥ 1:50	9.9% (95% CI 9.3–10.5)	Ranarum, Sejroe, Mini (highest among cattle MAT positives)	[32]
	Buffalo (water buffalo)	National cross-sectional survey	N = 1376 buffaloes	MAT; 24-serovar panel; cut-off ≥ 1:50	30.5% (95% CI 28.1–32.9)	Sejroe, Mini, Pomona	
	Pigs	National cross-sectional survey	N = 1898 pigs	MAT; 24-serovar panel; cut-off ≥ 1:50	10.8% (95% CI 9.5–12.3)	Ranarum, Pomona, Bratislava	
Thailand	Cattle, buffalo, pigs (passive surveillance)	Surveillance dataset (2010–2015)	N = 7218 (cattle 3648; buffalo 432; pigs 3138)	MAT; 23-serovar panel; cut-off ≥ 1:100	Cattle 28.1%; buffalo 24.8%; pigs 11.3%	Bratislava, Ranarum, Sejroe, Shermani, Tarassovi	[25]
Vietnam	Buffaloes	Multi-species cross-sectional (provincial) 2021	N = 1205 animals (multi-species) n = 52	MAT; 25-serovar panel; cut-off ≥ 1:100 (also reported at ≥1:200)	44.2%	Hebdomadis, Patoc, Castellonis, Javanica (Javanica predominant at higher cut-off)	[33]
	Cattle	Multi-species cross-sectional (provincial) 2021	n = 233	MAT; cut-off ≥ 1:100	24.9%	Diverse (15 serovars detected among cattle in study)	
	Pigs	Multi-species cross-sectional (provincial) 2021	n = 381	MAT; cut-off ≥ 1:100	10.2%	Javanica among serovars detected	
	Dogs, Cats, Rats	Multi-species cross-sectional (provincial) 2021	n = 219 (dogs) n = 164 (cats) n = 156 (rats)	MAT; cut-off ≥ 1:100	Dogs: 32.9%; cats: 12.2%; rats: 16.0%	Hebdomadis, Canicola/Javanica/others (varied)	
Malaysia	Cattle (selected subnational studies)	Multiple studies summarized in national review (1976–2023)	varied (example Kelantan n reported in original studies)	MAT (cut-offs variable: commonly 1:80–1:100 reported), some PCR and culture	Heterogenous: e.g., Kelantan 81.7% (one 2018 subnational survey) vs. other studies ~14%	Bataviae, Javanica, Ballum, Icterohaemorrhagiae, Canicola, Pomona, Hardjo/Hardjobovis	[29]
	Pigs	Selected PCR/serology	N = 81 (PCR) N = 869 (MAT)	PCR (lipL32/16S) and MAT cut-off 1:40	PCR: ~6%; MAT: 16.0%	Pomona	[29,35]
	Dogs and Cats	Multiple local studies synthesized (1976–2023)	Dog studies include working dogs, shelters, clinical cases; sample sizes variable	MAT (cut-offs variable; e.g., 1:80–1:100); some PCR on clinical cases/urine	Dogs: working dogs low (≈3–6%), shelters/diseased dogs high (up to ≈42.7% MAT and PCR in some studies); cats: ≈15–26% MAT in some reports	Bataviae, Javanica, Icterohaemorrhagiae, Canicola, Australis	[29,36,37]
	Rodents	Multiple local studies summarized	Sample sizes vary by study	PCR (lipL32), culture, MAT in some studies	PCR kidney prevalence reported in some studies up to >30–50% (study-dependent); culture/MAT also reported	Icterohaemorrhagiae, Bataviae, Javanica, and Ballum are commonly reported	[29]
Regional (meta-analysis)	Pigs (regional)	Meta-analysis of SEA studies (various years)	Pooled/compiled n across studies (N = 9219)	MAT studies heterogeneous (cut-offs 1:20–1:400; most use ≥1:100)	Reported pig seroprevalence ranged broadly across studies (8.2% to 73.4%)	Serogroups vary by country; panel composition affects detection	[34]
Regional (meta-analysis)	Rodents, cattle, dogs	Meta-analysis/regional reviews	Pooled/compiled n across studies (N = 9219)	MAT panels varied; PCR methods used in some studies	Rodents highly heterogeneous (6–>90% reported in global/regional compilations); cattle intermediate; dogs moderate	Icterohaemorrhagiae is often prevalent in rats; livestock serovars host-associated (e.g., Hardjo in cattle, Pomona in pigs)	[34]

**Table 3:** Climatic and environmental factors affecting leptospirosis transmission.

Factors	Consequence Affecting Leptospirosis Transmission	References
Rainfall	- Increased volume of contaminated water Increase in the population of rodents Heavy precipitation contributes to outbreaks And creates sustained wet environments for *Leptospira* survival	[12,15,70]
Temperature and humidity	- Creates optimal conditions for *Leptospira* sp. survival - Warm conditions support recreational water activities increasing risk of exposure	[12,14,71]
Seasonal pattern	- Outbreaks manifest during the rainy season - Coincides with agricultural calendars promoting increased contact to the pathogen	[12,14,72]
Land use	- Agricultural areas are conducive to rodent infestations e.g., rice paddy fields - Wet fields increase human–pathogen contact - Ineffective water management practices cause waterlogging	[73,74,75,76,77]
Vegetation cover	- Dense vegetation provides microclimatic conditions that support rodent populations - Forested areas are associated with higher leptospiral carriage in rodents	[78,79,80,81]
Urban settings	- Reduction in biodiversity, loss of habitat proliferation of wildlife in urban areas - Poor sanitation, informal infrastructure, frequent contact with floodwater	[82,83,84]
Rural settings	- Exposure to floodwaters - Agricultural practices encourage bacterial proliferation	[73,74,75]

## Data Availability

All data are available in the references provided.
